# Mesenchymal stem cell therapies for liver cirrhosis: MSCs as “conducting cells” for improvement of liver fibrosis and regeneration

**DOI:** 10.1186/s41232-019-0107-z

**Published:** 2019-09-09

**Authors:** Atsunori Tsuchiya, Suguru Takeuchi, Takayuki Watanabe, Tomoaki Yoshida, Shunsuke Nojiri, Masahiro Ogawa, Shuji Terai

**Affiliations:** Division of Gastroenterology and Hepatology, Graduate School of Medical and Dental Science, Niigata University, 1-757 Asahimachi-dori, Chuo-ku, Niigata, 951-8510 Japan

**Keywords:** Liver cirrhosis, Acute on chronic, Mesenchymal stem cell, Cell therapy

## Abstract

Mesenchymal stem cells (MSCs) can be cultured relatively easily and can be obtained not only from the bone marrow, but also from medical waste such as adipose tissue and umbilical cord tissue. Because of its low antigenicity, allogeneic MSC injection is safe. MSCs have been evaluated in more than 900 clinical trials in a variety of fields, with more than 50 clinical trials related to liver diseases. Experiments have suggested that MSCs function as “conducting cells” to affect various “effective cells” such as T cells, B cells, and macrophages. Recent clinical trials have focused on allogeneic MSCs. Thus, studies are needed to determine the most effective cell source, culture conditions, cell numbers, administration frequency, administration route, cost, safety, and liver disease treatments. Recently, the functions of exosomes have gained attention, and cell-free therapy may become possible as an alternative therapy for liver disease. In this review, we introduce general information, mechanism, representative clinical study data, recently started or planned clinical trials, and possibility of cell-free therapy of MSCs.

## Background

The liver, which is a vital organ, has many functions such as protein, triglyceride, cholesterol, and glycogen synthesis; detoxication; drug metabolism; and bile secretion, and has a high regenerative potential. However, the liver cannot withstand long-term chronic injury, severe acute injury, and acute on chronic injury. Chronic liver injuries such as those caused by hepatitis B virus (HBV) and hepatitis C virus infections, non-alcoholic steatohepatitis (NASH), and alcoholic liver injury have been widely studied. Because of the recent development of anti-viral drugs, it is easy to control HBV [1] and eradicate hepatitis C virus [2]. In contrast, the number of patients with NASH and alcoholic liver disease is increasing and no effective drugs except conventional alimentary therapy and exercise therapy are available; thus, development of new therapies for these diseases is becoming important [3]. Long-term damaged conditions gradually result in the loss of liver function and accumulation of extracellular matrix (ECM), finally leading to liver cirrhosis. Particularly, the prognosis of patients with decompensated liver cirrhosis is poor. Hepatic stellate cells are central players in liver fibrosis and the major precursors of activated myofibroblasts, which produce ECM during liver fibrosis [4, 5]. A recent study reported that fibrosis, rather than steatosis, determines the prognosis of patients [6, 7]. In acute liver injury such as hepatitis B, hepatitis A, and drug-induced liver injury, patients with excessive damage cannot be sufficiently treated by physicians. Additionally, acute on chronic liver failure (ACLF), the definition of which slightly differs by region [8–11], is also a recently focused disease condition. Recently proposed diagnostic criteria for ACLF in Japan include “patients with cirrhosis and a Child–Pugh score of 5–9 should be diagnosed as having ACLF when a deterioration of liver function (serum bilirubin level ≥5.0 mg/dL and prothrombin time value ≤40% of the standardized values and/or international normalization rate ≥1.5) caused by severe liver damage develops within 28 days after acute insults, such as alcohol abuse, bacterial infection, gastrointestinal bleeding, or the exacerbation of underlying liver diseases” [11]. These chronic, acute, and acute on chronic liver injuries can cause death. Although liver transplantation can be conducted, the shortage of donor organs is a serious problem. Cell therapies may be useful for treating these diseases. We firstly showed that autologous bone marrow cell infusion (ABMi) therapy was effective for the decompensation of liver cirrhosis patients [12]. Several clinical cases in this study [13] have shown that bone marrow-derived cells can improve liver fibrosis and subsequently improve liver function. Therefore, recently, we shifted our focus to cell therapies using mesenchymal stem cells. Mesenchymal stem cells (MSCs) have been widely examined in clinical trials to evaluate their safety and effectiveness in improving liver fibrosis and liver function. Positive results have been observed in numerous studies of animal models [14–17].

In this review, we describe the general information of MSCs, mechanisms of MSC therapies (i.e., the conducting effect of MSCs), recently published outcomes of MSC therapy, clinical trials that have recently started or will begin soon, and recent research trends using extracellular vesicles obtained from MSCs.

## General information of MSCs

MSCs have been used in many fields to treat a variety of diseases such as neural, heart, liver, intestinal, and lung diseases. According to ClinicalTrials.Gov, more than 900 clinical trials have been registered in a variety of fields and are increasing by nearly 100 trials each year. MSCs can be obtained not only from the bone marrow, but also from medical waste such as umbilical cord tissue, adipose tissue, amniotic tissue, and dental pulp. These cells are relatively easy to expand, maintain, and cryopreserve, while maintaining their viability. MSCs are positive for the common markers CD73, CD90, and CD105 and show differential potential towards adipocytes, osteoblasts, and chondroblasts under appropriate conditions. The cells are used to replace damaged cells or tissues mainly in the orthopedic field; however, their main functions are determined by trophic factors including chemokines, cytokines, growth factors, and exosomes, and MSCs exhibit anti-inflammation, anti-oxidant, angiogenesis, and anti-fibrosis effects [4, 5]. Of these functions, MSCs are most commonly applied to achieve anti-inflammation effects. MSCs produce various factors such as nitric oxide/indoleamine 2,3-dioxygenase, interleukin (IL)-10, tumor necrosis factor-inducible gene-6, and prostaglandin E2; inhibit T cell activation and expansion; induce regulatory T cells; alter the polarity of macrophages to the anti-inflammatory phenotype; and control the function of dendritic cells, B cells, and natural killer cells [18, 19]. Another important characteristic of MSCs is that they generally have low immunogenicity. MSCs express low or modest levels of major histocompatibility complex class I molecules and lack expression of major histocompatibility complex class II and co-stimulatory molecules, such as CD40, CD80, and CD86 (B7–2), leading to low immunogenicity, suggesting that MSCs can avoid immune responses in recipients; thus, injection of autologous or allogenic MSCs has been employed in clinical studies. For example, Lalu et al. performed a meta-analysis of the safety of MSCs in clinical trials and found that autologous and allogenic MSC therapies are related to transient fever but not related to infusion toxicity, organ system complications, infection, death, and malignancies [20]. Allogenic MSC therapy has the potential to be applied in many patients.

## MSCs function as “conducting cells” in liver disease

**Fig. 1:**
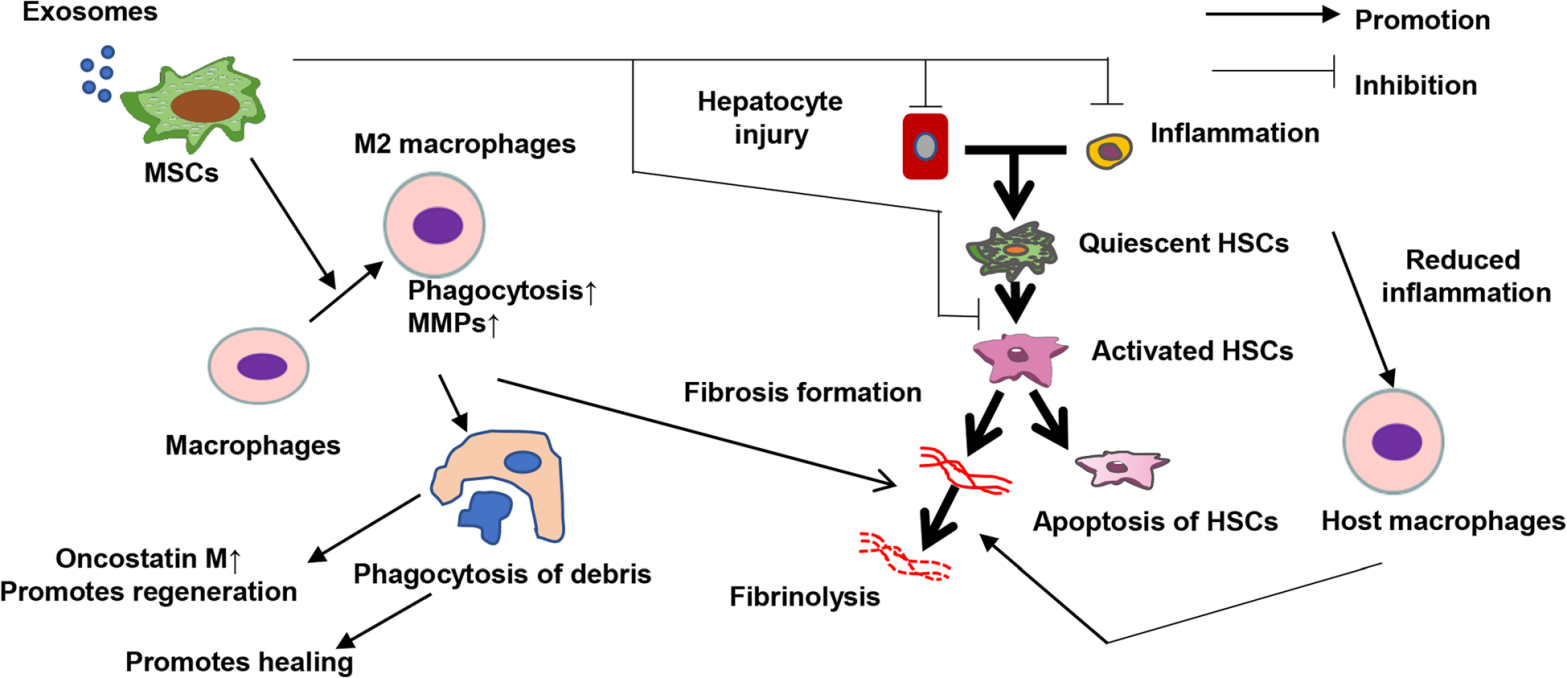
Mechanisms of MSCs for liver disease. MSCs have various effects including the reduction of hepatocyte injury and inflammation. Additionally, MSCs affect macrophages and increase matrix metalloproteinase expression and phagocytosis, promoting the regenerative process

The mechanisms of MSCs for treating liver diseases have been evaluated from various perspectives in basic studies. MSCs have anti-inflammatory effects and reduce damages to hepatocytes [21]. These anti-inflammatory effects and decreases in hepatocyte damage reduce the activation of hepatic stellate cells [22] and direct the effects of MSCs to reduce hepatic stellate cell activation [23]. Additionally, we recently reported the effects against macrophages. MSCs change the polarity of macrophages towards an anti-inflammatory phenotype, increase the production of matrix metalloproteinases to reduce the ECM, and increase the ability of phagocytosis of hepatocyte debris (during this process, macrophages increase the levels of pro-regenerative factors) (Fig. 1) [14]. When we administered bone marrow-derived MSCs with macrophages produced by culturing bone marrow cells for 7 days, host macrophages and neutrophils were also recruited to the liver.

The behaviors of macrophages after administration showed differences between studies. Some studies reported that administered MSCs can migrate to and function in the liver [24]. However, our recent studies of a liver cirrhosis mouse model using two-photon excitation microscopy revealed that when bone marrow-derived macrophages (green fluorescent protein-labeled) and MSCs (DsRed-labeled) were administered at the same time via the tail vein, most MSCs migrated to the lung and a low percentage of MSCs migrated to the liver, which disappeared from both the lung and liver after 7 days. In contrast, macrophages migrated to both the lung and liver, where they remained for 7 days. Particularly, in the liver, administered macrophages migrated to the damaged area where excess ECM and hepatocyte debris were detected. Furthermore, we observed that the administered GFP-positive macrophages phagocytosed the debris in hepatocytes in the liver [14].

While some aspects of the mechanisms of MSCs remain unclear, these results revealed that MSCs function indirectly as “conducting cells,” while macrophages, T cells, B cells, and other cells function directly as “effective cells.”

## Summary of MSC therapies from recently published papers

Many reports have been published describing the results of clinical trials using MSCs. Zhao et al. reported a meta-analysis of previously published papers up to June 2017. They evaluated 23 reports of studies comparing MSC therapy to conventional treatment. The authors concluded that MSC-based therapy is relatively safe and improved liver function during the first 6 months after administration. A single injection administration via the hepatic artery and MSCs derived from the bone marrow are optimal in terms of improving liver function [25]. The analyzed cases were quite heterogenous and included papers only written in Chinese; thus, next, we show two representative studies.

Suk et al. reported a phase II study using bone marrow-derived MSCs for treating alcoholic liver cirrhosis. An MSC culture from 10 to 20 ml of bone marrow aspirated 1 month before the first injection was administered one or two times (5 × 10^7^ cells/time) via the hepatic artery and compared to the control. Results of biopsy performed 6 months after cell administration revealed 25% (one-time cell administration) and 37% (two administrations) reductions in the fibrosis area. Furthermore, the Child-Pugh scores of both the single and double administration groups were improved significantly at 12 months after cell injection [26].

Lin et al. reported an open-label non-blinded randomized controlled study using 1.0–10 × 10^5^ cells/kg of allogeneic bone marrow cells for treating patients with HBV-related ACLF once per week for 4 weeks; these subjects were followed for 24 weeks. The clinical laboratory results showed that serum total bilirubin and model for end-stage liver disease scores were improved compared to those obtained after standard medical therapy. The authors further observed that the incidences of severe infection and mortality from multiple organ failure were reduced after cell administration [27].

Two representative cases of cirrhosis and ACLF of MSC therapy were shown above, both of which were treated safely and showed some favorable effects. Based on these studies, additional clinical studies were designed. The next section describes recently designed clinical trials.

## Recently started or planned clinical trials

**Fig. 2:**
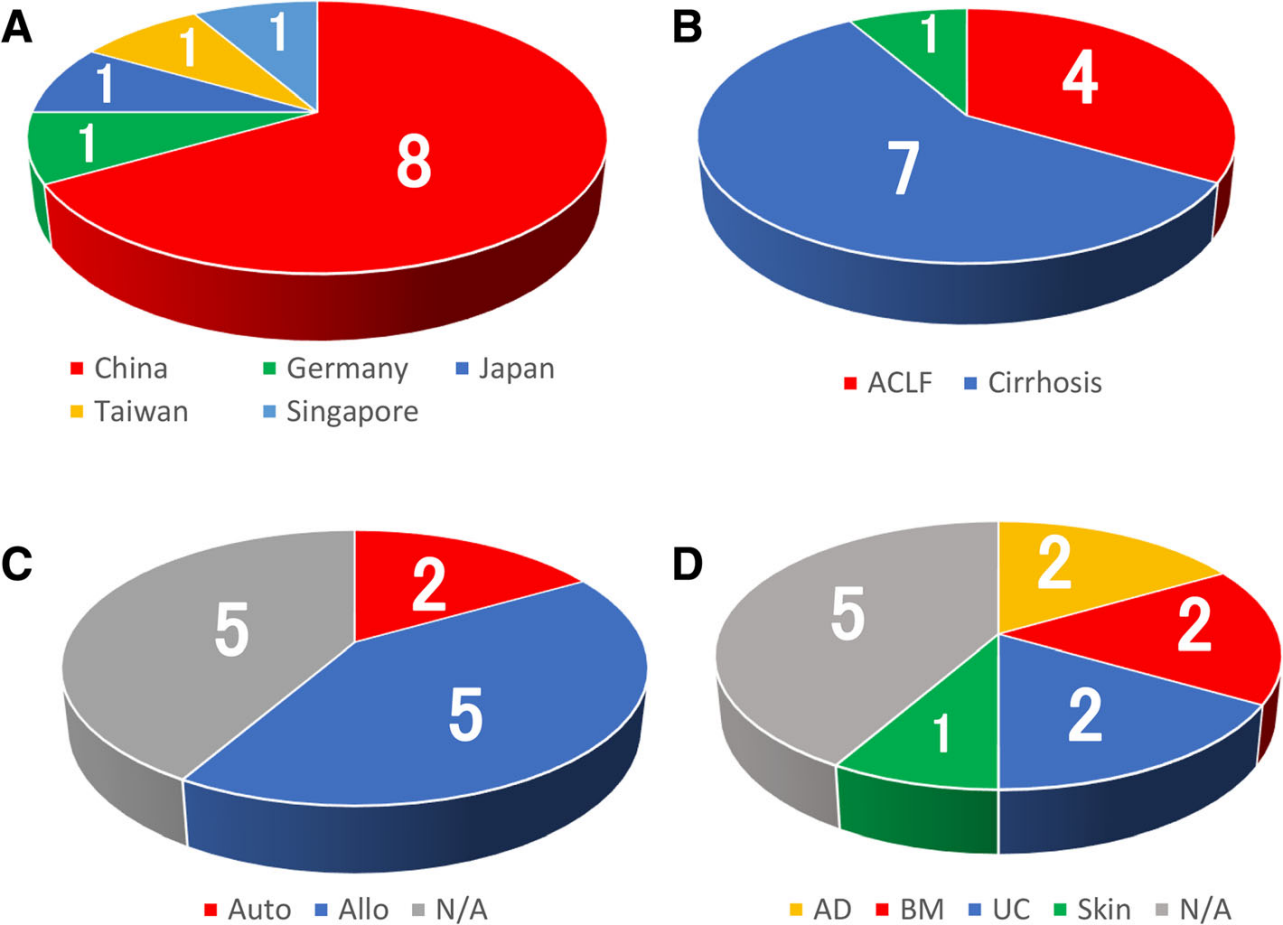
Recent trends in clinical trials using MSCs. Proportion of country (**a**), disease conditions (**b**), autologous or allogeneic (**c**), and tissue origin of MSCs (**d**) in recent clinical trials

To describe recent trends in clinical trials using MSCs, we evaluated clinical studies which began or will begin after 1 January 2017 according to ClinicalTrials.gov. Thirteen clinical trials were registered; 1 case was a follow-up study of a clinical trial, and thus, we excluded this case and analyzed the other 12 cases. As shown in Tables 1, 8 of 12 (66.7%) cases were from China and 1 case (8.3%) each occurred in Germany, Japan, Taiwan, and Singapore (Fig. 2a). Four of 12 cases (33.3%) were ACLF, 7 of 12 cases (58.3%) were cirrhosis in which each study’s etiology of cirrhosis slightly differed, and 1 case (8.3%) was a target for primary biliary cholangitis (Fig. 2b). Five of 12 (41.6%) cases were treated with allogeneic MSCs, and 2 of 12 cases were treated with autologous MSCs (16.7%). In 5 of 12 cases, it was unclear whether allogeneic or autologous cells were used; however, based on the study design and disease condition, allogeneic MSCs were administered in most cases, suggesting that allogeneic cases were increased compared to previously reported frequencies (allogeneic, 53%; autologous, 45%) (Fig. 2c). Regarding the cell origin, 1 case (8.3%) occurred in the skin, while the other cases occurred in the bone marrow (2 cases; 16.7%), adipose tissue (2 cases; 16.7%), and umbilical cord tissue (2 cases; 16.7%). The cases of bone marrow origin were all autologous cases (Fig. 2d). The cell numbers employed in the trials slightly differed; approximately 0.1–1.0 × 10^6^ cells/kg were injected 1–4 times, cell administration in 10 of the 12 cases (83.3%) was performed via the peripheral vein, and 1 case (8.3%) involved autologous bone marrow case from the hepatic artery, suggesting that the recent trend in administration is the peripheral vein. However, further studies of direct infusion of MSCs are necessary to achieve efficient effects. All clinical trials are still in phase I or/and II.

**Table 1:** Recently started or planned clinical trials

	Country	Conditions	Auto/Allo	Origin	Cell number	Cell injection times	Route	Phase	Study design
1	China	ACLF	N/A	N/A	1–10 × 10^5^/kg	4	Peripheral vein	I/II	Randomized/open label
2	China	ACLF	N/A	N/A	0.1–1 × 10^6^/kg	3	Peripheral vein	N/A	Randomized/double blind
3	Germany	ACLF	Allo	Skin (ABCB5+ cells)	2 × 10^6^/kg	3	Peripheral vein	I/II	Non-randomized/open label
4	China	Cirrhosis	N/A	N/A	N/A	N/A	N/A	I/II	Non-randomized/open label
5	China	Cirrhosis	Allo	UC	1.5 × 10^6^/kg	2~4	Peripheral vein	II	Non-randomized/open label
6	China	Cirrhosis (HBV, HCV)	N/A	N/A	1 × 10^6^/kg	4	Peripheral vein	N/A	Randomized/single blind
7	Japan	Cirrhosis (NASH, HCV)	Allo	AD	N/A	1	Peripheral vein	I/II	Non-randomized/open label
8	Taiwan	ACLF	Allo	AD	0.5–2 × 10^6^/kg	N/A	Peripheral vein	I	Non-randomized/open label
9	Singapore	Cirrhosis	Auto	BM	0.5–1 × 10^6^/kg	N/A	Peripheral vein	I/II	Non-randomized/open label
10	China	Alcoholic liver cirrhosis	Auto	BM	5 × 10^7^ cells/10 ml	1	Hepatic artery	I	Non-randomized/open label
11	China	Cirrhosis (HBV)	Allo	UC	6 × 10^7^ (30 ml)	N/A	Peripheral vein	I	Non-randomized/open label
12	China	PBC	N/A	N/A	0.1–1 × 10^6^/kg	3	Peripheral vein	N/A	Randomized/double blind

## Recent research trend of using extracellular vesicles obtained from MSCs

Although most MSCs were trapped in the lung, they showed therapeutic effects. To explain this phenomenon, extracellular vesicles (EVs) have been evaluated [28–34]. EVs include apoptotic bodies (50–4000 nm in diameter), microvesicles (MVs; 100–1000 nm in diameter), and exosomes (40–100 nm in diameter), among which exosomes are the most widely studied [31]. Exosomes are lipid vesicles produced by multivesicular bodies prior to extracellular secretion. They can be sedimented by ultra-centrifugation and include endosome-derived components as well as many bioactive molecules such as proteins, lipids, mRNAs, microRNAs (miRNAs), long non-coding RNAs, transfer RNA, genomic DNA, cDNA, and mitochondrial DNA [35]. Exosome membranes are enriched in cholesterol, sphingomyelin, ceramide, and lipid raft proteins. Exosomes are cell type-specific; however, they contain evolutionarily conserved sets of proteins including tetraspanins (CD81, CD63, and CD9), heat shock proteins (HSP60, HSP70, and HSP90), AlIX, and tumor susceptibility gene 101 and have been reported to have multiple functions including angiogenesis, cell proliferation, and collagen reduction. MSCs are relatively easy to expand and well-known to produce abundant exosomes and thus are theoretically ideal tools for developing cell-free therapies [28, 30–32, 34]. Haga et al. reported that mouse bone marrow-derived MSC EVs have therapeutic effects for hepatic failure induced by d-galactosamine (d-gal) and tumor necrosis factor α. In this experiment, high levels of EVs were observed after 6 h in the liver and spleen. Jiang et al. reported that exosomes derived from human umbilical cord-derived MSCs alleviate acute liver failure (lipopolysaccharide/d-galactosamine-induced liver injury model) [36]. They reported that MSC exosomes reduced the activation of the NLRP3 inflammasome, IL-1β, and IL-6 in macrophages. Borrelli et al. reported the use of drug-loaded EVs for treating hepatocellular carcinoma. Exosomes contain diverse and numerous miRNAs, and thus, determining the roles of miRNA is very difficult. Ferguson et al. reported that most previous studies used a candidate approach with specific miRNAs to assess their therapeutic effects; however, this approach may not fully capture the various biological effects induced by miRNAs in the MSC exosomes of recipient cells [29]. System-level studies are thus needed.

## Conclusions

MSCs are attractive cell therapies that function as “conducting cells” against many types of immune cells and induce a variety of therapeutic effects. However, several years have passed since the first MSC theories were postulated, and thus, these clinical studies must be evaluated to determine whether MSC therapies are indeed effective in human liver diseases. In recent clinical trials, the trend of MSC therapy appears to have shifted from administration of autologous cells towards allogeneic cells. This research area is very attractive for developing effective anti-fibrotic therapies. Studies are needed to determine the most effective cell source, culture condition, cell number, administration frequency, and administration route, at low cost for treating specific liver diseases. Cell-free therapy using exosomes is an attractive approach.

## Data Availability

All data generated or analyzed during this study are included in this published article.

## Abbreviations

ACLF: Acute on chronic liver failure
ECM: Extracellular matrix
EV: Extracellular vesicle
MSC: Mesenchymal stem cell
